# Riboswitch-controlled lipid conversion enables functional membrane asymmetry in artificial cells

**DOI:** 10.1038/s42003-026-09890-7

**Published:** 2026-03-19

**Authors:** Koki Kamiya, Sumin Lee, Kotaro Baba

**Affiliations:** Graduate School of Science and Technology, Gunma University, Gunma, Japan

**Keywords:** Biophysical chemistry, Synthetic biology, Membrane biophysics

## Abstract

Dynamic regulation of lipid membrane composition is fundamental to living cells; however, synthetic analogs capable of such regulation remain scarce. Here, we present an artificial cell platform in which riboswitch-mediated expression of phospholipase D (PLD) enables stimulus-responsive lipid remodeling within lipid vesicles. In this system, chemically induced activation of a fluoride-responsive riboswitch triggers cell-free synthesis of PLD, which hydrolyzes phosphatidylcholine (PC) to phosphatidic acid (PA) in the inner leaflet of the vesicles. This enzymatic reaction generates a negatively charged asymmetric membrane, enabling functionalization with mechanosensitive channels such as the mechanosensitive channel of large conductance (MscL). We characterized the kinetics of asymmetry generation by varying plasmid DNA and fluoride concentrations, and evaluated membrane behavior using lipid compositions with or without cholesterol. Our platform demonstrates a strategy for coupling gene expression to dynamic membrane remodeling and underscores the potential of riboswitch-regulated lipid transitions in building environment-responsive artificial cells with programmable functions.

## Abstract (web summary)

Dynamically generating asymmetric lipid vesicles are formed by a fluoride-responsive riboswitchmediated expression of phospholipase D, which hydrolyzes phosphatidylcholine to phosphatidic acid in the inner leaflet of the vesicles.

## Introduction

Modulating cellular functions is crucial for regulating gene expression, signal transduction, metabolic activity, and intercellular communication. Synthetic biology enables the construction of artificial systems that mimic such regulatory mechanisms, particularly through riboswitches—engineered RNA elements that control gene expression in response to specific small molecules such as coenzymes, nucleotides, amino acids, and ions^1–3^. Riboswitches have been successfully applied to modulate T cell function^4^, intracellular metabolic pathways^5–7^, and intercellular signaling^8^.

Artificial cells based on lipid vesicles containing riboswitch DNA and cell-free transcription–translation systems offer a promising platform for chemically regulated gene expression and protein synthesis^9–15^. These vesicles can respond to external stimuli that diffuse inward and trigger riboswitch-mediated synthesis of functional proteins, enabling programmable responses to environmental cues such as reagents and heat. For instance, the transport of histamine into the lipid vesicles containing a histamine-responsive riboswitch and a cell-free protein synthesis (CFPS) system activates the riboswitch to express α-hemolysin (α-HL) within the lipid vesicles. The pore-forming α-HL transports fluorescence probes from the inner phase to the outer phase owing to the α-HL proteins that are reconstituted into the lipid bilayer of the lipid vesicles^11^. Using a thermo-responsive riboswitch, the α-HL proteins are expressed at 42 °C of incubation and transport calcein to the lipid vesicles^14^.

In parallel, the lipid composition of cell membranes—particularly their asymmetric distribution between inner and outer leaflets—plays essential roles in processes such as apoptosis and signal transduction^16–19^. Several methods exist to construct asymmetric lipid vesicles that mimic eukaryotic membranes^20–30^. These asymmetric lipid vesicles facilitate the observation of lipid dynamics (flip-flop, bending rigidity, and microdomain)^23,31–34^, reconstitution and function of membrane proteins^23,35^, transport of water-soluble proteins^36,37^, and delivery of mRNA and protein to living cells^38^. These studies were performed on the asymmetric lipid vesicles that mostly lack dynamic control over lipid composition. Enabling stimulus-responsive transitions in lipid asymmetry would provide valuable insights into biological membrane dynamics and facilitate functional reconstitution in artificial cells. This gradual induction of lipid asymmetry enables real-time monitoring of membrane protein functions and other biological functions governed by lipid asymmetry, providing new insights into their underlying mechanisms and facilitating the construction of stimulus-responsive synthetic cells incorporating biomolecules regulated by membrane asymmetry.

**Fig. 1:**
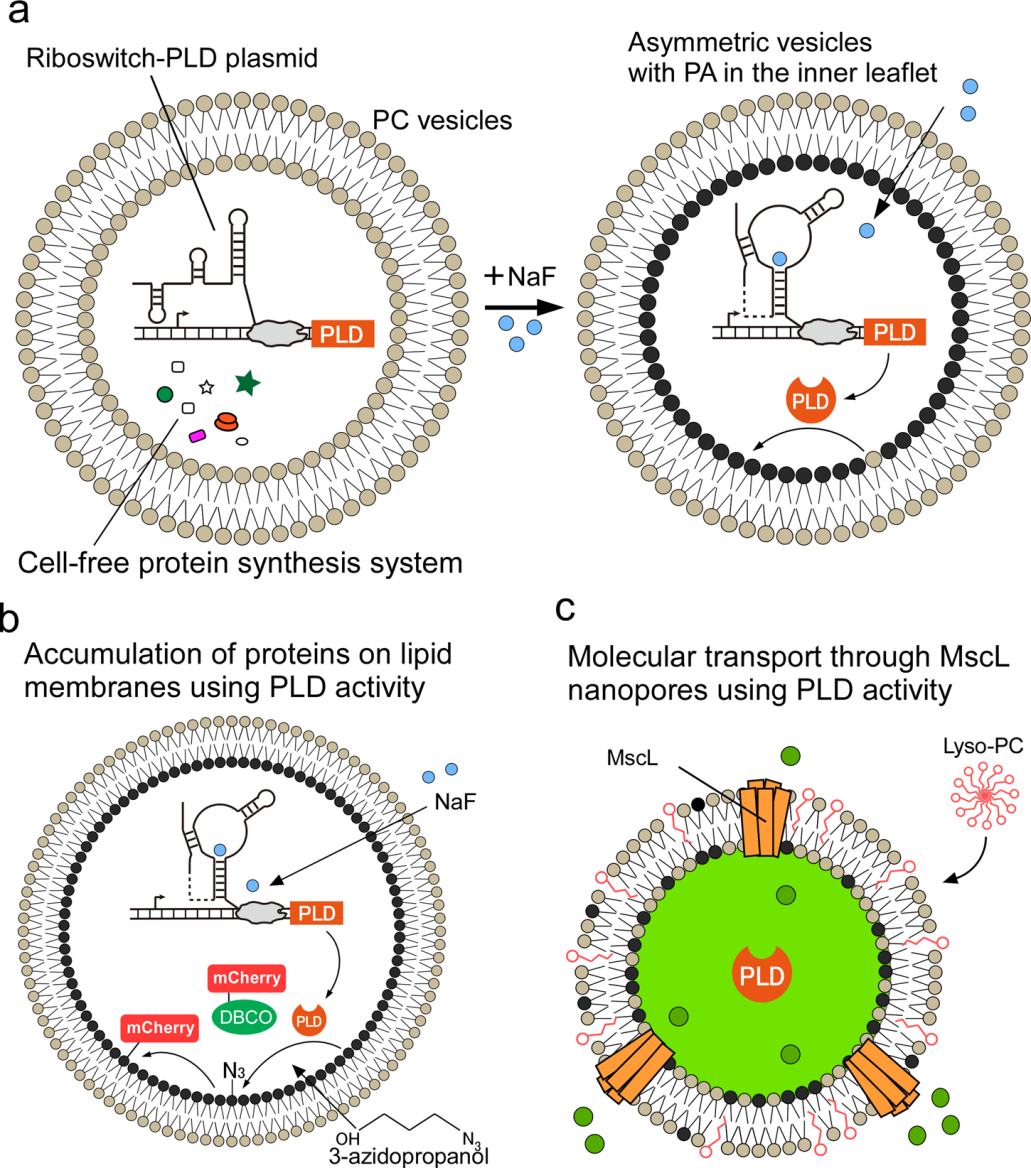
Schematic of dynamically generating asymmetric lipid vesicles in response to chemical stimuli via the activity of phospholipase D (PLD). **a** Generation of asymmetric lipid vesicles with phosphatidic acid (PA). **b** Accumulation of proteins on the lipid membranes using the transphosphatidylation activity of PLD, which is riboswitch-regulated. **c** Molecular transport through MscL nanopores on the PA membranes is generated by the activity of PLD, which is riboswitch-regulated expression.

Here, we present a strategy for dynamically generating asymmetric lipid vesicles in response to chemical stimuli via phospholipase D (PLD), which converts phosphatidylcholine (PC) into phosphatidic acid (PA) (Fig. 1a). PLD enzymes catalyze the hydrolysis of PC to generate choline and PA and the transphosphatidylation of PC and alcohol substrates to generate choline and phosphatidyl alcohol products^39,40^. By coupling riboswitch-regulated expression of PLD with a cell-free system encapsulated within symmetric vesicles, we achieved the formation of asymmetric lipid vesicles via in situ enzymatic remodeling of the inner membrane leaflet. We selected a sodium fluoride (NaF) riboswitch because the protein synthesis induced by the riboswitch does not require the addition or synthesis of Lacl proteins, and NaF can freely diffuse across the lipid membrane and does not interfere with cell-free protein synthesis^41^. Next, fluorescence proteins (mCherry) were accumulated on the inner leaflet of the lipid vesicles using the transphosphatidylation activity of PLD (Fig. 1b). Finally, the molecular transport of MscL was activated on the lipid vesicle membrane, where negatively charged lipids were generated by PLD-catalyzed hydrolysis in response to external stimuli (Fig. 1c). This controlled lipid transition facilitates membrane functionalization via charge-sensitive elements such as mechanosensitive channels and enables environment-responsive behavior in artificial cell models.

## Results

### Enzyme activity of phospholipase D synthesized via a cell-free protein synthesis system

**Fig. 2:**
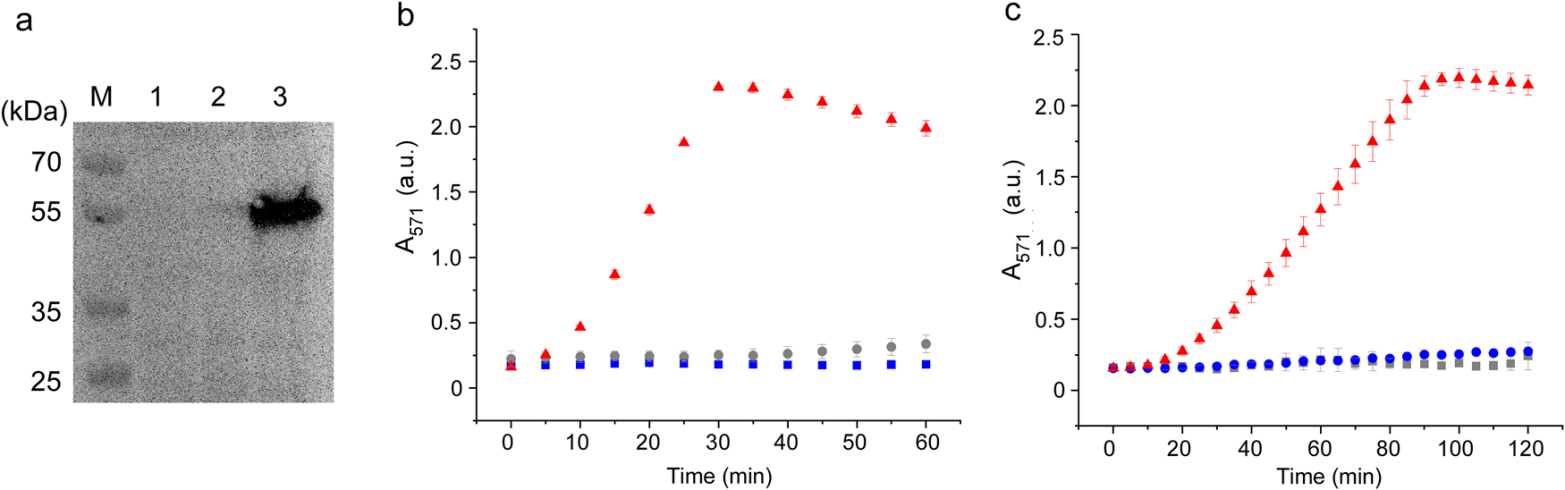
Synthesis and function of PLD in buffer solution. **a** Western blot analysis of PLD synthesized by S30 CFPS system. M: protein marker; Lane 1: only S30 CFPS solution (−DNA), Lane 2: wild-type PLD; Lane 3: mutant PLD. **b** Enzymatic activity assay of *Escherichia coli* S30 lysate-synthesized PLD, measuring choline hydrolysis (orenge: mutant PLD; gray: wild-type PLD; blue: −DNA control) (*N* = 3 experiments). **c** Enzymatic activity assay of mutant PLD synthesized under control of a fluoride-responsive riboswitch, measuring choline hydrolysis (orenge: +DNA and +NaF; gray: −DNA and +NaF; blue: +DNA and −NaF) (*N* = 3 experiments). Final concentration of PLD plasmid DNA was 20 ng/µL. PLD phospholipase D, NaF sodium fluoride, A_571_ absorbance at 571 nm.

We focused on a mutant form of PLD that exhibits higher enzymatic activity than the wild-type form to enable rapid conversion of PC to PA within vesicles. This mutant PLD, previously reported by Tei et al.^42^, was synthesized using a cell-free protein synthesis (CFPS) system (*Escherichia coli* S30 extract). Western blot analysis confirmed the synthesis of mutant PLD after 2 h of incubation at 37 °C, with a prominent band observed at approximately 55 kDa, indicating correct expression by the CFPS system (Fig. 2a and Supplementary Fig. 1). In contrast, the synthesis of wild-type PLD was not confirmed, although wild-type PLD was synthesized using PUREfrex 2.0 (Supplementary Fig. 2). Therefore, the DNA sequence of wild-type PLD may not be compatible with the S30 CFPS system. We used the S30 CFPS system to control protein synthesis under a fluoride-responsive riboswitch. We next assessed the enzyme activity of the synthesized mutant PLD using a resorufin-based assay. Resorufin is produced when PC is hydrolyzed by PLD, releasing choline. An increase in the absorbance of resorufin was detected starting at 10 min and peaked at 30 min (Fig. 2b), after that the absorbance decreased because resorufin was further oxidized by HRP and H_2_O_2_ into colorless and non-fluorescent products^43^.

In contrast, no significant absorbance change was observed in samples expressing wild-type PLD-encoded plasmid DNA or in the absence of PLD-encoded plasmid DNA. These results demonstrate that the mutant PLD synthesized using the CFPS system retains enzymatic activity to catalyze PC hydrolysis, generating PA and choline. We used this mutant PLD in the subsequent experiments.

The synthesis and activity of PLD under the control of a fluoride-responsive riboswitch were investigated. A PLD expression cassette was constructed by inserting the PLD coding sequence downstream of the related fluoride riboswitch in a plasmid vector^44^. Reactions of the CFPS system with or without the riboswitch-PLD plasmid (final concentration: 20 ng/µL) were prepared and supplemented with 3 mM sodium fluoride (NaF). Reactions were incubated at 37 °C for 4 h, and PLD activity was quantified with a resorufin-based assay (Fig. 2c). The absorbance of resorufin in the reacted solutions containing both plasmid DNA and NaF gradually began to increase at approximately 20 min and continued to increase throughout the measurement period. By contrast, reactions lacking plasmid DNA (but containing NaF) showed no change in absorbance. When plasmid DNA was present without NaF, resorufin absorption was detectable but markedly lower than in the NaF-induced condition, indicating low-level, leaky PLD expression. To investigate the suppression of non-specific PLD synthesis, we repeated the assay using different concentrations of plasmid DNA (final concentrations: 2, 4, and 10 ng/µL) (Supplementary Fig. 3). At 10 ng/µL, the difference in resorufin production between plasmid DNA samples in the presence and absence of NaF was maximal, demonstrating that PLD activity in the CFPS system can be controlled by selecting an appropriate combination of plasmid DNA and NaF concentration.

### Conversion of PC to PA inside lipid vesicles using PLD activity

**Fig. 3:**
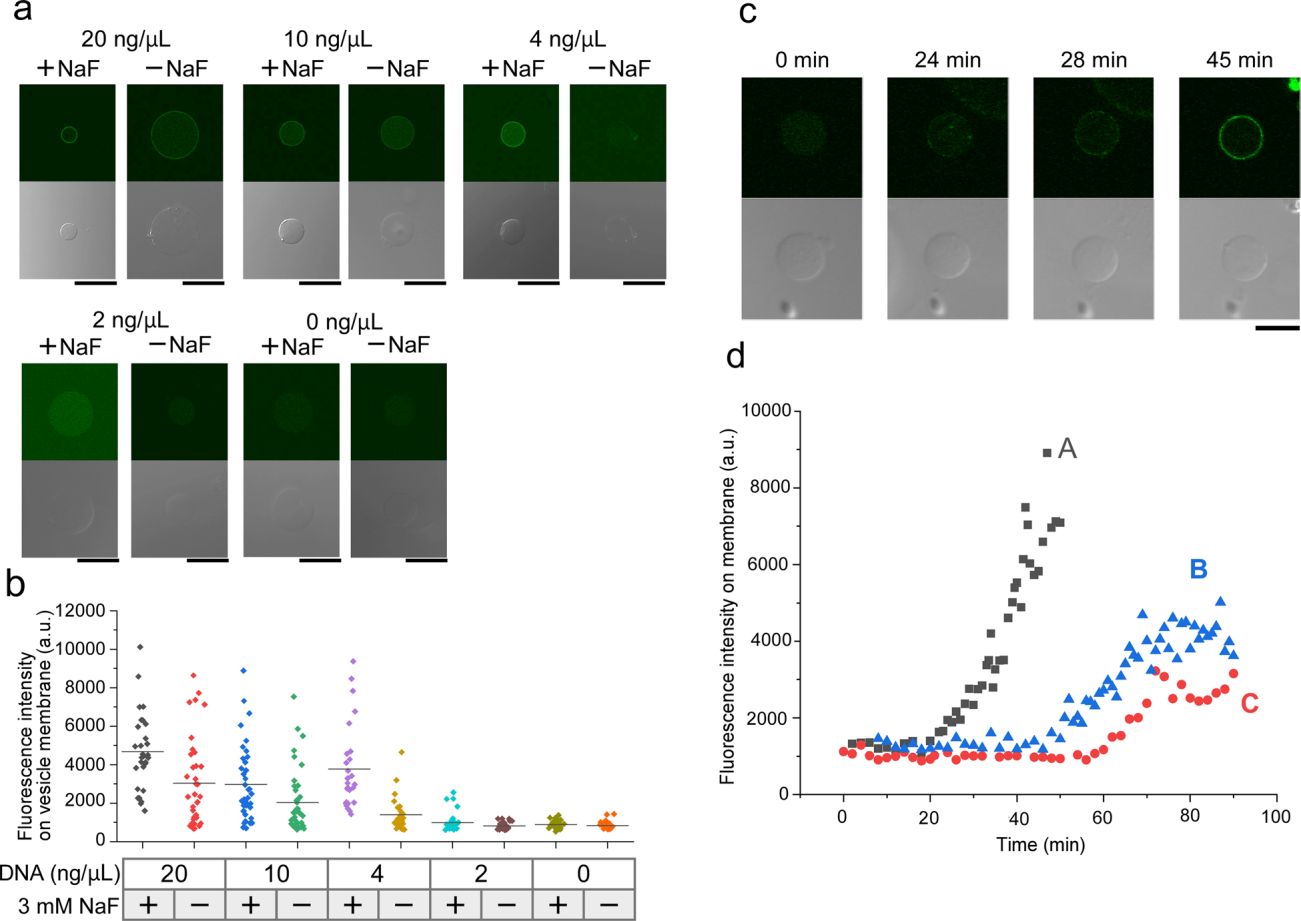
Synthesis and function of PLD in lipid vesicles. **a** Confocal laser scanning microscopy images of spo20-GFP localization on lipid vesicles with or without 3 mM NaF at final plasmid DNA concentrations of 20, 10, 4, 2, and 0 ng/µL after 1 h incubation at 37 °C. Scale bar: 10 µm. **b** Quantification of Spo20-GFP fluorescence intensity on lipid vesicles under the same conditions (after 1 h incubation at 37 °C) as in **a**. Specifically, vesicles with 20 ng/µL plasmid DNA: +NaF (*n* = 29 vesicles, *N* = 3 experiments) or −NaF (*n* = 36 vesicles, *N* = 3 experiments), 10 ng/µL plasmid DNA: +NaF (*n* = 38 vesicles, *N* = 3 experiments) or −NaF (*n* = 39 vesicles, *N* = 3 experiments), 4 ng/µL plasmid DNA: +NaF (*n* = 26 vesicles, *N* = 3 experiments) or −NaF (*n* = 29 vesicles, *N* = 3 experiments), 2 ng/µL plasmid DNA: +NaF (*n* = 37 vesicles, *N* = 3 experiments) or −NaF (*n* = 36 vesicles, *N* = 3 experiments), and 0 ng/µL plasmid DNA: +NaF (*n* = 28 vesicles, *N* = 3 experiments) or −NaF (*n* = 28 vesicles, *N* = 3 experiments) were analyzed. Black bars indicate the average fluorescence intensity for each group. **c** Time-lapse confocal images showing Spo20-GFP localization on lipid vesicles containing 3 mM NaF and 4 ng/µL plasmid DNA. Scale bar: 10 µm. **d** Time-course of Spo20-GFP fluorescence intensity on lipid vesicle. A, B, and C indicate the fluorescence intensity at each vesicle. PLD phospholipase D, spo20-GFP green fluorescent protein-tagged Spo20 lipid-binding domain, NaF sodium fluoride.

To control the expression and activity of PLD within dioleoyl‑phosphatidylcholine (DOPC) vesicles, we investigated the conversion of PA from PC by varying the concentrations of PLD plasmid DNA containing the fluoride riboswitch (2, 4, 10, or 20 ng/µL). Cell-free protein synthesis solution, the riboswitch-PLD plasmid, and PA sensor Green Fluorescent Protein-tagged Spo20 lipid-binding domain (Spo20-GFP)^45,46^ were co-encapsulated in DOPC vesicles. After 60 min at 37 °C, vesicles were imaged using CLSM in the presence or absence of 3 mM NaF (Fig. 3a). The fluoride riboswitch retained full functionality when encapsulated within the lipid vesicles. At 2 ng/µL plasmid DNA, the fluorescence intensity of Spo20-GFP on the DOPC vesicle membrane was negligible, regardless of NaF. With 4 ng/µL plasmid DNA, fluorescence intensity of Spo20-GFP on the vesicle membrane increased markedly in the presence of NaF, confirming the generation of PA from PC by NaF-dependent PLD activity. A vesicle was scored “PA‑positive” when its membrane fluorescence exceeded the highest value observed in negative‑control vesicles lacking plasmid DNA (Figs. 3b and S4). The percentages of vesicles with Spo20-GFP fluorescence on their membrane at 20, 10, 4, and 2 ng/µL PLD plasmid DNA were 100% (*n* = 29/29; negative control [−NaF]: 63.9%, *n* = 23/36), 78.9% (n = 30/38; −NaF: 51.3%, *n* = 20/39), 96.2% (*n* = 25/26; −NaF: 24.1%, *n* = 7/29), and 10.8% (*n* = 4/37; −NaF: 0%, *n* = 0/36), respectively. At plasmid concentrations below 2 ng/µL, few vesicles exhibited Spo20-GFP fluorescence on the membrane, even in the presence of NaF, indicating that PLD expression was insufficient at this DNA level. Conversely, when the concentration of PLD plasmid DNA in the vesicles exceeded 10 ng/µL, Spo20-GFP fluorescence was abundant both with and without NaF, suggesting that the concentration of the PLD plasmid DNA was high. The largest difference between the numbers of vesicles with Spo20-GFP fluorescence in the presence or absence of NaF occurred at 4 ng/µL of the PLD plasmid DNA. At this concentration, the fluorescence intensity showed a significant difference between the vesicles with and without NaF (*P* < 0.01; Fig. 3b). Therefore, we selected 4 ng/µL as the optimal concentration of the PLD plasmid DNA for subsequent experiments. This optimal concentration of the PLD plasmid DNA required inside the lipid vesicles was lower than that in the bulk solution (Supplementary Fig. 3). Previous studies have shown that compartmentalization of the CFPS system leads to different transcription and translation rates compared to the bulk CFPS system, owing to the lipid membrane that allows the exchange of membrane-permeable small molecules such as amino acids between the lipid vesicle and the external environment^47^. Moreover, we attempted to normalize the fluorescence intensity on the membranes by the diameter of each lipid vesicle. No correlation was observed between the vesicle diameter and fluorescence intensity on the membranes. In our previous study, transcription and translation inside giant lipid vesicles prepared by the droplet transfer method were successfully carried out using the S30 CFPS system. Also, no correlation was observed between the vesicular area and the fluorescence intensity of transcription and translation. This variation in fluorescence intensity may be caused by factors other than vesicle size, such as variability in the amount of CFPS solution and plasmid DNA in the w/o emulsions, the membrane-permeable small molecular exchange between the outer and inner solutions across the membrane, differences in the transcription and translation inside lipid vesicles, and differences in the membrane thickness^48,49^. This study was performed by the lipid vesicles with 5–20 µm diameter.

We next monitored the time-dependent conversion of the vesicle membrane composition from PC to PA via PLD activity. Time-lapse images of DOPC vesicles encapsulating the CFPS solution, PLD plasmid DNA, and Spo20-GFP were obtained after adding 3 mM NaF to the outer solution and incubating the samples at 37 °C (Fig. 3c). Spo20-GFP fluorescence first appeared on the membrane after approximately 30–50 min and increased gradually until plateauing at approximately 70–80 min (Fig. 3d). A previous study investigated time-dependent changes (30, 60, 90, and 120 min) in transcriptional and translational activities in the DOPC vesicles. It reported that transcription begins by 30 min and translation by 60 min^50^. We used superPLD with catalytic efficiencies up to 100-fold higher than that of wild-type PLD^42^. Therefore, the observed PC to PA conversion time aligns with these expression timelines. Finally, we estimated the PA fraction in the vesicular membrane using a calibration curve to convert Spo20-GFP fluorescence intensities to PA percentages (Supplementary Figs. 5 and S6). The PA percentages in DOPC vesicles at maximum Spo20-GFP fluorescence intensity were 21.4, 13.6, and 15.8 mol%.

### Formation of vesicles with asymmetric lipid composition

**Fig. 4:**
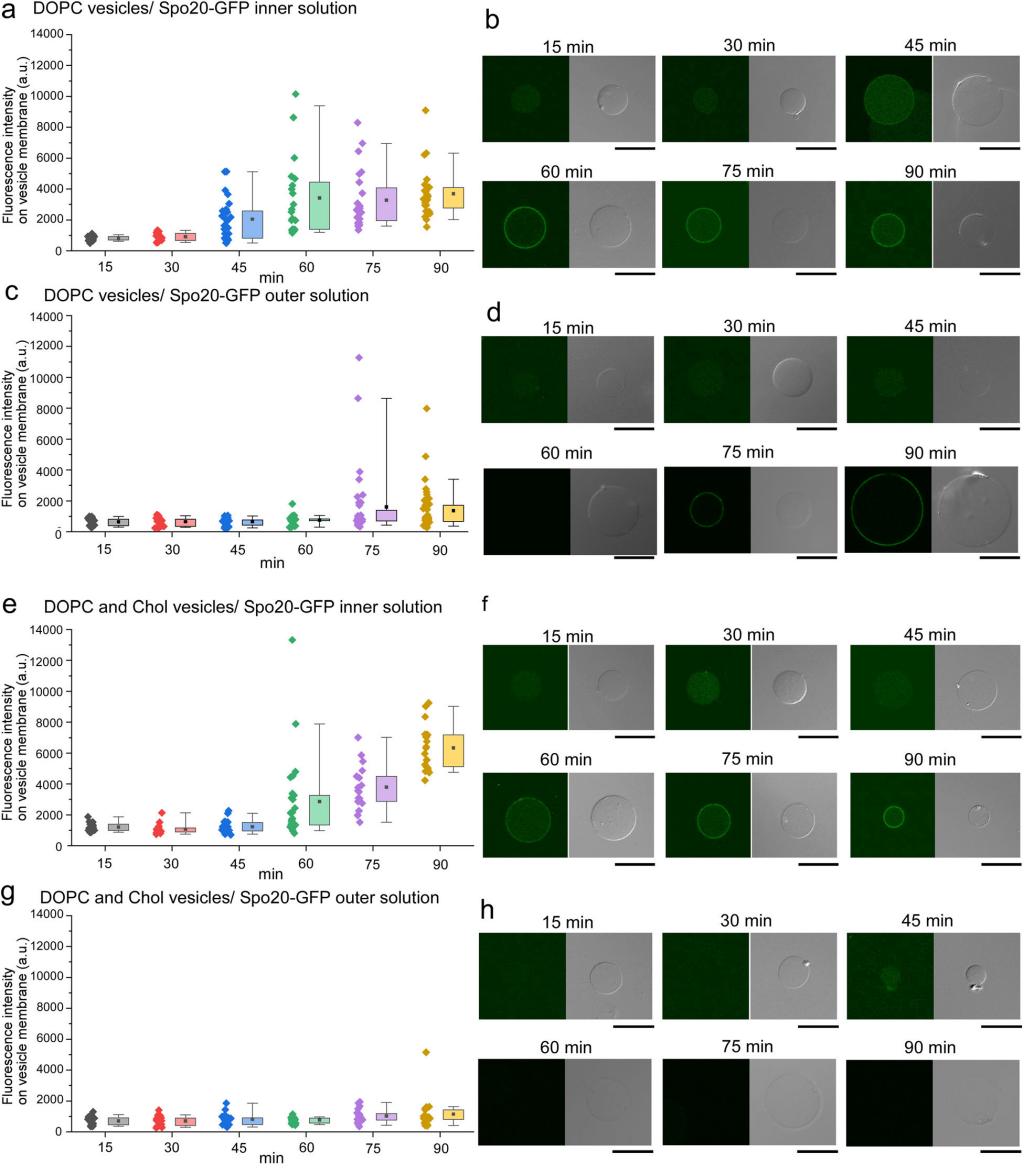
Formation of asymmetric lipid vesicles activated by PLD activity. Scatter‑plus‑box plots of Spo20‑GFP fluorescence intensity on DOPC vesicles at different time points after NaF addition, when Spo20-GFP is present in the inner solution (**a**) (15 min (*n* = 23 vesicles, *N* = 3 experiments), 30 min (*n* = 20 vesicles, *N* = 3 experiments), 45 min (*n* = 25 vesicles, *N* = 3 experiments), 60 min (*n* = 20 vesicles, *N* = 3 experiments), 75 min (*n* = 24 vesicles, *N* = 3 experiments), and 90 min (*n* = 28 vesicles, *N* = 3 experiments)); and outer solution (**c**) (15 min (*n* = 28 vesicles, *N* = 3 experiments), 30 min (*n* = 28 vesicles, *N* = 3 experiments), 45 min (*n* = 27 vesicles, *N* = 3 experiments), 60 min (*n* = 29 vesicles, *N* = 3 experiments), 75 min (*n* = 36 vesicles, *N* = 3 experiments), and 90 min (*n* = 41 vesicles, *N* = 3 experiments)). Representative confocal fluorescence micrographs corresponding to the plotted data for inner solution (**b**); and outer solution (**d**) at each time. Scale bar: 10 µm. Formation of asymmetric lipid vesicles activated by PLD activity. Scatter‑plus‑box plots of Spo20‑GFP fluorescence on cholesterol-containing vesicles at different time points, when Spo20-GFP is present in the inner solution (**e**) (15 min (*n* = 15 vesicles, *N* = 3 experiments), 30 min (*n* = 18 vesicles, *N* = 3 experiments), 45 min (*n* = 21 vesicles, *N* = 3 experiments), 60 min (*n* = 25 vesicles, *N* = 3 experiments), 75 min (*n* = 17 vesicles, N = 3 experiments), and 90 min (*n* = 21 vesicles, *N* = 3 experiments)); and outer solution (**g**) (15 min (*n* = 21 vesicles, *N* = 3 experiments), 30 min (*n* = 21 vesicles, *N* = 3 experiments), 45 min (*n* = 18 vesicles, *N* = 3 experiments), 60 min (*n* = 21 vesicles, *N* = 3 experiments), 75 min (*n* = 20 vesicles, *N* = 3 experiments), and 90 min (*n* = 23 vesicles, *N* = 3 experiments)). Representative confocal fluorescence micrographs corresponding to the plotted data for inner solution (**f**); and outer solution (**h**) at each time. Scale bar: 10 µm. Spo20‑GFP green fluorescent protein-tagged Spo20 lipid-binding domain, DOPC dioleoyl‑phosphatidylcholine, NaF sodium fluoride, Chol cholesterol.

We investigated whether activating PLD inside lipid vesicles could generate asymmetric lipid composition in the membrane. To visualize the distribution of dioleoyl‑phosphatidic acid (DOPA) on DOPC vesicle membranes, we added the DOPA-binding probe Spo20-GFP either to the inner or outer solution of the vesicles. Fluorescence in the inner solution indicates the existence of DOPA in the inner leaflet, whereas fluorescence in the outer solution indicates DOPA in the outer leaflet. DOPC vesicles containing CFPS solution and PLD plasmid DNA, in the presence of Spo20-GFP in the inner or outer solution of the vesicles, were incubated for 15, 30, 45, 60, 75, and 90 min at 37 °C after adding 3 mM NaF in the outer solution. When Spo20-GFP was inside the vesicles, membrane fluorescence became detectable at 45 min and increased steadily thereafter, both in intensity and in the number of fluorescent vesicles (Fig. 4a, b). In contrast, when Spo20-GFP was only in the outer solution, little membrane fluorescence was seen up to 60 min; signal and vesicle counts began to rise at 75 min (Fig. 4c, d).

Vesicles displaying membrane-associated Spo20-GFP were defined as those whose fluorescence intensity exceeded the mean intensity after 15 min of incubation plus three standard deviations (+3σ). When Spo20-GFP was present in the inner solution, the percentages of vesicles with membrane fluorescence after 15, 30, 45, 60, 75, and 90 min were 0% (0/23), 10% (2/20), 64% (16/25), 90% (18/20), 100% (24/24), and 100% (28/28), respectively (Fig. 4a). In contrast, when Spo20-GFP was added to the outer solution, the corresponding percentages were 0% (0/28), 0% (0/28), 0% (0/27), 3.44% (1/29), 25% (9/36), and 31.7% (13/41) (Fig. 4c). The difference between the numbers of vesicles with Spo20-GFP fluorescence in the inner and outer leaflets was greatest 45 and 60 min after NaF addition, with fluorescence intensities significantly higher for the inner leaflets at both time points (*P* < 0.01). These results show that 45–60 min after the addition of NaF, PLD encapsulated inside the vesicles generated a pronounced asymmetric distribution of DOPA in the DOPC membrane. Over longer times, newly formed DOPA in the inner leaflet gradually translocated to the outer leaflet, yielding symmetric DOPC/DOPA vesicles. This redistribution is attributed to the high fluidity of the DOPC vesicle membranes and the smaller head group of PA relative to PC.

Next, we investigated the effect of adding 30 mol% cholesterol—which lowers membrane fluidity—on the dynamic formation of an asymmetric DOPA distribution in the vesicles. Previous studies show that NaF permeates vesicle membranes containing this level of cholesterol. In the presence of Spo20-GFP in the inner solution, membrane-associated fluorescence first became detectable at 60 min; thereafter, both the fluorescence intensity and the vesicle count gradually increased (Fig. 4e, f). This delayed onset relative to cholesterol-free DOPC vesicles reflects the reduced fluidity and permeability of the vesicle membranes imparted by 30 mol% cholesterol. By 75 min, the fluorescence intensity of all cholesterol-containing vesicles exceeded that of vesicles lacking cholesterol. In the presence of Spo20-GFP in the outer solution, no membrane fluorescence appeared during the first 60 min; only a few labeled vesicles were detected at 75 min and 90 min (Fig. 4g, h). In contrast to cholesterol-free vesicles, the outer leaflet fluorescence intensity remained low throughout, suggesting that incorporation of PA into the lipid bilayer is prevented by the decreased membrane fluidity. Therefore, DOPC vesicles containing cholesterol show high-efficiency generation of PA asymmetry, retaining PA in the inner leaflet. When Spo20-GFP was added to the inner solution, the fractions of vesicles exhibiting membrane fluorescence after incubation for 15, 30, 45, 60, 75, and 90 min were 0% (0/15), 5.6% (1/18), 9.5% (2/21), 12% (3/25), 88.2% (15/17), and 100% (21/21), respectively (Fig. 4e). In contrast, when Spo20-GFP was added to the outer solution, the corresponding vesicle fractions were 0% (0/21), 0% (0/21), 5.6% (1/18), 0% (0/21), 15% (3/20), and 21.7% (5/23) (Fig. 4g). The disparity between the numbers of vesicles with Spo20-GFP fluorescence in the inner or outer leaflets became more pronounced at 60 min and 75 min after the addition of NaF. Fluorescence intensities were significantly higher for inner-loaded vesicles at 75 min and 90 min (*P* < 0.01 for both). Relative to cholesterol-free DOPC vesicles, those containing cholesterol displayed higher Spo20-GFP fluorescence intensity in the inner leaflet and lower fluorescence in the outer leaflet at 75 min and 90 min. By 90 min, the inner-leaflet fluorescence of cholesterol-containing vesicles clustered at high values, whereas cholesterol-free vesicles showed broader, low intensity distributions.

Moreover, we estimated the amount of DOPA in the outer leaflet (DOPA translocation from the inner leaflet to the outer leaflet) on the lipid vesicles (Supplementary Table 1) from the fluorescence inteinsites (Fig. 4a, c, e, g) and the calibration curve (Supplementary Fig. 6). In DOPC vesicles (Fig. 4a, c), the amount of DOPA in the inner leaflet after 90 min of incubation ranged from 11.5 to 19.3 mol%, while the amount of DOPA in the outer leaflet ranged from 10.7 to 14.6 mol%. Among the vesicles analyzed, 26 out of 27 vesicles exhibited the inner leaflet DOPA contents in the range of 11.5–19.3 mol%, while 9 out of 39 vesicles showed the outer leaflet DOPA contents between 10.7 and 14.6 mol%. In DOPC vesicles containing cholesterol (Fig. 4e, g), the amount of DOPA in the inner leaflet after 90 min of incubation ranged from 16.9 to 22.7 mol%, while the amount of DOPA in the outer leaflet contained up to 10.4 mol% DOPA. All analyzed vesicles (20/20) exhibited the inner leaflet DOPA contents in the range of 16.9–22.7 mol%, while only 1 out of 22 vesicles showed the outer leaflet DOPA contents between 10.0 and 10.4 mol%. The amount of DOPA in the outer leaflet and the number of vesicles containing more than 10 mol% DOPA in the outer leaflet were lower in the cholesterol-containing vesicles than in the vesicles without cholesterol. Therefore, the flip-flop rate is faster in the vesicles without cholesterol than in the cholesterol-containing vesicles. A flop rate (transfer of PA from the inner leaflet to the outer leaflet) in the vesicles without cholesterol was roughly estimated from the molar ratio of DOPA in the outer leaflet (10.7 and 14.6 mol%), which was detected in 9 out of 39 vesicles. In the case of the vesicles with a diameter of 10 µm^51^, the flop rate was 8.64 × 10^3^ to 1.18 × 10^4^ lipids/sec. These results indicate that cholesterol-containing vesicles generate and maintain a pronounced PA asymmetry after PLD-mediated hydrolysis. Reduced membrane fluidity in cholesterol-containing vesicle membranes impedes the translocation of PA from the inner to the outer leaflet and limits leakage of components of the CFPS.

### Accumulation of water-soluble proteins on artificial lipid membranes

**Fig. 5:**
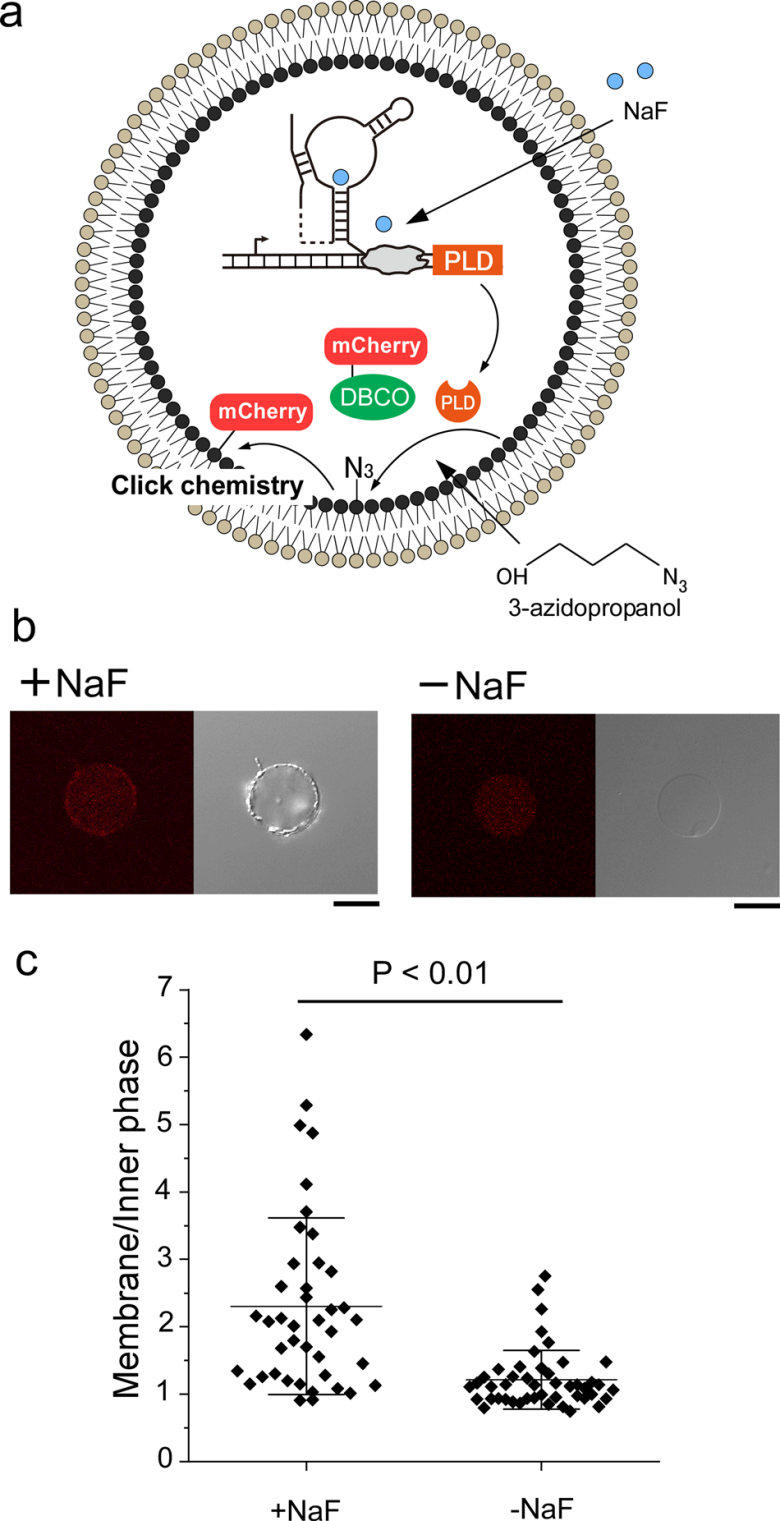
PLD-driven accumulation of mCherry on lipid vesicles via NaF-controlled riboswitch. **a** Schematic illustration of mCherry accumulation on the inner leaflet of lipid vesicles containing plasmid DNA and DBCO-conjugated mCherry upon addition of 3 mM NaF and 3-azidopropanol to the outer solution. **b** Confocal laser scanning microscopy images of mCherry fluorescence on lipid vesicles in the presence or absence of 3 mM NaF. Scale bar: 10 µm. **c** Quantification of membrane fluorescence intensity in lipid vesicles in the presence (*n* = 41 vesicles, *N* = 3 experiments) or absence (*n* = 46 vesicles, *N* = 3 experiments) of 3 mM NaF, normalized to the fluorescence intensity of the inner phase. Statistical significance was defined as *P* < 0.01. PLD phospholipase D, NaF sodium fluoride, DBCO Dibenzocyclooctyne.

In a previous report, PC head groups in the living cells were modified into azido-lipids when reacted with Clickable Alcohols using the transphosphatidylation activity of PLD^39,40^. The chemical fluorescent probe accumulated on the cell membrane in the inner leaflet via click chemistry between azido-lipids and the alkyne-chemical fluorescent probe. We tested whether this system of protein accumulation could be applied to recruit proteins to artificial cell membranes (Fig. 5a). Cell-sized DOPC vesicles, encapsulating the CFPS system solution, PLD plasmid DNA, and alkyne-conjugated mCherry (Supplementary Fig. 7), were prepared by the droplet-transfer method. Azido-propanol was added to the outer solution of the cell-sized vesicles. After 60 min of incubation in the presence and absence of NaF, the vesicles were observed by confocal microscopy (Fig. 5b). Pronounced mcherry fluorescence was detected on the membranes of NaF-treated vesicles, whereas only weak labeling was seen without NaF. The percentages of vesicles whose membrane fluorescence intensity exceeded 1.5-fold background were 66% (27/41) with NaF and 13% (6/46) without NaF (Fig. 5c); the difference was statistically significant. These results demonstrate that the stimulus-dependent recruitment of proteins to vesicular membranes is enabled by the transphosphatidylation activity of PLD, which is controllable through an NaF-responsive riboswitch. This protein accumulation method offers a platform for regulating protein reactions to facilitate the production of materials and changes in membrane morphology in artificial cells.

### Enhancement of membrane protein activity by converting neutral lipid membranes to negatively charged membranes

**Fig. 6:**
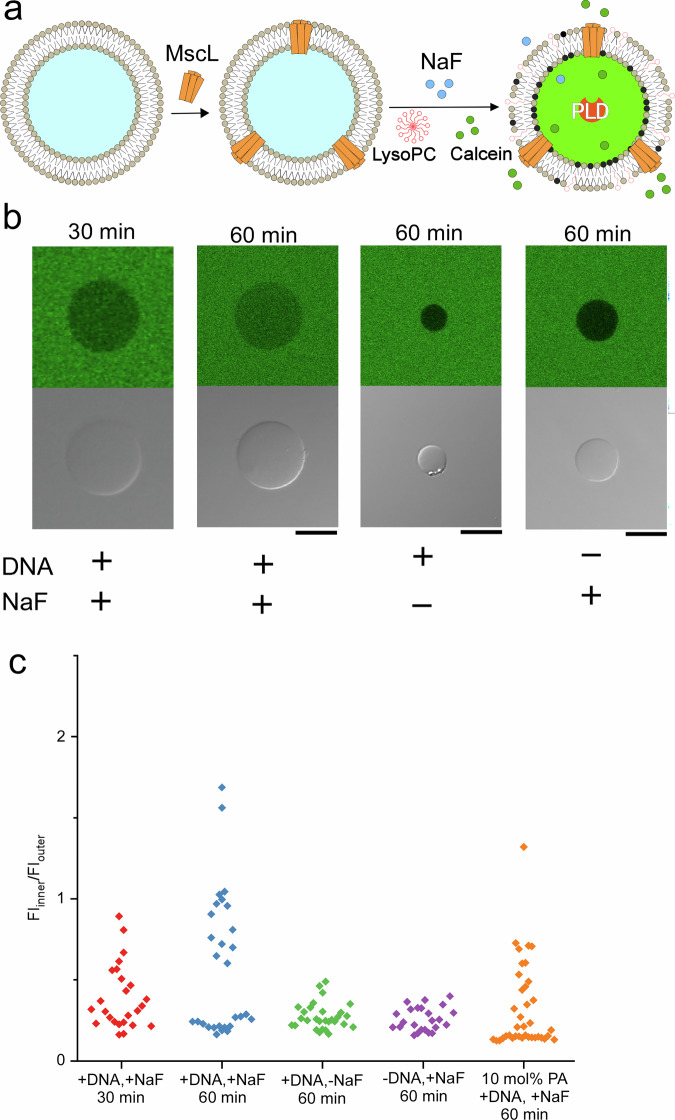
MscL-mediated transport of calcein into lipid vesicles via PA lipids activated by PLD expressed through a NaF-responsive riboswitch. **a** Schematic illustration of calcein transport into lipid vesicles via MscL nanopores, triggered by the addition of NaF and lyso-PC. **b** Confocal laser scanning microscopy fluorescence images of calcein-containing lipid vesicles under four experimental conditions. Scale bar: 10 µm. **c** Fluorescence intensities of calcein in asymmetric lipid vesicles after incubation for 30 or 60 min under the following conditions: with plasmid DNA and NaF at 30 min (red; *n* = 25 vesicles, *N* = 2 experiments), with plasmid DNA and NaF at 60 min (blue; *n* = 29 vesicles, *N* = 2 experiments), with plasmid DNA only, no NaF (green; *n* = 29 vesicles, *N* = 2 experiments), and with NaF only, no plasmid DNA (purple; *n* = 24 vesicles, *N* = 2 experiments). A fluorescence intensity of calcein in asymmetric lipid vesicles containing 10 mol% PA in the inner leaflet (with plasmid DNA and NaF) after incubation for 60 min (orange; *n* = 38 vesicles, *N* = 2 experiments). MscL mechanosensitive channel of large conductance, PA phosphatidic acid, PLD phospholipase D, NaF sodium fluoride, lyso-PC lyso-phosphatidylcholine.

We modulated membrane protein activity by increasing the fraction of negatively charged lipids generated through PLD-catalyzed hydrolysis triggered by external stimuli. To demonstrate the concept, we integrated PLD hydrolysis into the mechanosensitive channel of large conductance (MscL) reconstituted in DOPC vesicles (Fig. 6a). MscL forms pentameric nanopores that transport small molecules (≤10 kDa) by sensing the negatively charged lipids and membrane tension^52,53^. Reconstitution of MscL into vesicles containing PLD activity was investigated by CLSM. Alexa Fluor 633-conjugated MscL localized to the DOPC vesicle surface, and its surface concentration was 37.8 ± 18.4 μM regardless of plasmid DNA inclusion—consistent with previous reports using vesicles containing CFPS solution, DNA, and NaF (Supplementary Fig. 8).

We next monitored molecular transport through MscL nanopores in vesicles containing the PLD hydrolysis system. MscL nanopores in the lipid membranes are activated in the presence of negatively-charged phospholipids and Lyso-PC^52^. MscL nanopores-reconstituted vesicles encapsulating the CFPS system solution, and PLD plasmid DNA were prepared. When NaF and lyso-PC were added to the outer solution of the vesicles, PA generated in the membrane activated MscL channels, allowing transportation of calcein into the vesicles. Thus, chemical stimuli controlled molecular transport in this system. We examined the MscL-reconstituted vesicles by CLMS under four experimental conditions (Fig. 6b) and measured the fluorescence intensity with each vesicle’s inner phase and in the outer phase, FI_inner_ and FI_outer_, respectively. For MscL-vesicles containing both DNA and NaF (+DNA/+NaF) after 60 min of incubation, fluorescence intensities fell into two groups: a FI_inner_/FI_outer_ ratio of over 0.5 and below 0.5 (Fig. 6c). Vesicles with fluorescence intensities greater than 0.5 accounted for 48% (14/29). This plot clearly reveals the extent of molecular transport into the vesicles, with some vesicles in the positive control reaching a ratio of 1. Ratios exceeding 1 indicate that calcein became concentrated within the vesicles. In contrast, after 60 min of incubation, the number of +DNA/–NaF vesicles and –DNA/+NaF vesicles exceeding this threshold were 0% (0/29) and 0% (0/24), respectively. After 30 min, 28% (7/25) of +DNA/+NaF vesicles had intensities over 0.5. In addition, the molecular transport through MscL nanopores in vesicles containing 10 mol% PA in the inner leaflet was investigated. After 60 min of incubation, the vesicles with fluorescence intensities over 0.5 accounted for 21% (8/38). These results indicate that transport of calcein into the lipid vesicles began within 30 min of incubation, and the proportion of vesicles having a ratio of over 0.5 increased with time. Time-lapse imaging showed PA formation starting approximately 20 min after incubation (Fig. 3c, d), consistent with the onset of calcein transport. In our experiment of PA conversions in the DOPC vesicles (Figs. 3d and 4), PA reached 11–22.6 mol% in the inner leaflet of DOPC vesicles after 60 min. Because MscL nanopores are reported to open when negatively charged lipids exceed approximately 5 mol%, the observed PA levels are sufficient to activate the pores^54^. From these results, two populations of the fluorescence intensity in the +DNA/+NaF vesicles after 60 min of incubation may be caused by a broad range of PA conversion in the lipid membranes and low efficiency of the molecular transport through MscL nanopores in the vesicles containing 10 mol% PA in the inner leaflet. The C-terminus of MscL interacts with negatively charged phospholipids to open the pore. In this study, MscL was randomly oriented within the vesicular membrane, thereby reducing its functionality. Collectively, external chemical stimulation generated enough PA to open MscL nanopores and thereby enable regulated molecular transport.

## Discussion

In living cells, biological processes are regulated by changes in gene expression and protein–protein interactions that respond to external cues. Artificial cell membranes, such as lipid vesicles and liposomes, are therefore attractive platforms for biomedical applications, cell therapy, material production, and studies on the origin of life^38,55–57^. Recently, researchers have begun to mimic the programmed reactions of living cells within lipid vesicles. By encapsulating riboswitch DNA together with a CFPS system, these vesicles can regulate various cell functions, controlling the expression of both membrane-bound and water-soluble proteins in response to external stimuli such as specific chemical reagents or temperature changes^11,14^. To the best of our knowledge, we are the first to demonstrate the formation of cell-sized asymmetric lipid vesicles through chemically induced transitions in lipid composition.

To date, dynamic transitions in the lipid composition on the inner leaflet of cell-sized synthetic lipid vesicles have not yet been demonstrated, although asymmetric lipid vesicles can be formed by dynamically exchanging lipids in the outer leaflet by methyl-β-cyclodextrin (mbCD)^27,58^. Here, we show that in situ lipid remodeling of vesicle membranes activates membrane-protein functions such as molecular transport. PA is generated from PC through the hydrolytic activity of PLD (Fig. 4). Using this platform, asymmetric lipid vesicles with complex head-group compositions, including serine- and ethanolamine-containing lipids, will be generated. These dynamic transitions to complex asymmetric lipid vesicles—mimicking the plasma membranes of eukaryotic cells—provide an opportunity to uncover previously unrecognized biological phenomena. Because lipid remodeling can be monitored in real time by time-lapse imaging, our lipid vesicles can help understand the initiation of these novel biological processes and reactions. Moreover, the platform can be programmed to respond to a wide range of external stimuli, enabling the construction of artificial cells that sense and adapt to their environment by modifying their functions. Thus, our platform supports the development of diverse artificial cell systems by leveraging stimulus-responsive membrane asymmetry as a key design principle.

Furthermore, the accumulation of fluorescent water-soluble proteins on the inner leaflet of the lipid vesicles was induced by external stimuli (Fig. 5). Because these proteins are closely packed, such accumulation contributes to the efficiency of enzyme-cascade reactions activated by the same stimuli. Artificial cell models that respond to cancer cell-derived stimuli by initiating intravesicular enzymatic production of therapeutic agents thus hold promise for future development.

The decrease in membrane fluidity caused by adding cholesterol to vesicle membranes contributed to maintaining the asymmetric lipid distribution. Our results show that lipid vesicles containing 30 mol% cholesterol retained their asymmetry for 90 min. After 90 min, the average fluorescence intensity of the inner leaflet in cholesterol-rich vesicles remained significantly higher than that of cholesterol-free vesicles, whereas the fluorescence intensity of the outer leaflet was unchanged from its value at 15 min of incubation. These results indicate that the converted asymmetric lipid vesicles are sufficiently stable and reproducible for long-term observation of biochemical reactions, providing a valuable platform for dynamic analysis of artificial cells. Given that eukaryotic plasma membranes also contain approximately 30 mol% cholesterol, vesicles with this composition represent a highly suitable model of the native membrane environment for artificial cell studies^59^.

We demonstrated the activation of molecular transport through MscL nanopores by the conversion of PC to PA via PLD controlled by riboswitch. Following the addition of NaF for 30 min, calcein transport was detected in the lipid vesicles, whereas negative-control vesicles showed no calcein flux even after the addition of NaF for 60 min. The platform therefore exhibits both swift responsiveness and high precision, suggesting its potential for developing synthetic cells that respond to diverse external stimuli—including signals from living cells—by triggering accurate and programmable internal responses.

In our system, approximately 20 mol% of PC was converted to PA within 90 min. Although long-term incubation would further increase the PA concentration, complete conversion is difficult, as the small PA headgroup promotes high intrinsic curvature and ultimately compromises vesicle stability at elevated PA concentrations (Supplementary Fig. 9). Notably, negatively-charged phospholipids in the inner leaflet of the plasma membrane account for approximately 30 mol%^60^, indicating that full conversion is not required to achieve a physiologically relevant level of membrane asymmetry. Because PLD activity cannot be externally inhibited in this system, PA production proceeds continuously. To impose an upper limit on PA accumulation, a concentration-dependent choline-responsive riboswitch can be encapsulated within the lipid vesicles. Choline, produced stoichiometrically during the PLD-mediated conversion of PC to PA, provides an intrinsic signal that can activate the riboswitch. Following NaF addition, PLD synthesis is initiated and PA production begins. Once choline reaches the activation threshold, the riboswitch induces the synthesis of proteases and RNases, thereby digesting PLD and its mRNA and establishing negative feedback that stabilizes the PA concentration within the lipid vesicles.

Our results further show that dynamic changes in lipid composition stimulated by external stimuli activate membrane-embedded proteins to mediate both molecular transport and accumulation of water-soluble proteins on the inner leaflet. By coupling these processes, this platform orchestrates complex intravesicular reactions. Consequently, synthetic cells equipped with this system could detect biological signals released from cancer cells and enzymatically produce therapeutic agents within the vesicle compartments, paving the way for the development of self-regulated artificial cells for biomedical applications.

## Materials and methods

1,2-Dioleoyl-*sn*-glycero-3-phosphocoline (DOPC) and 1-palmitoyl-2-hydroxy-*sn*-glycero-3-phosphocholine (16:0 LysoPC) were purchased from Avanti Polar Lipids Inc. (Alabaster, AL). 2-amino-2-hydroxymethyl-1, 3-propanediol (Tris), 2-[4-(2-hydroxyethyl)-1-piperazinyl] ethanesulfonic acid (HEPES), NaCl, N,N-dimethyldodecylamine N-oxide (LDAO), imidazole, glucose, sucrose, sodium hydrosulfite, proteinase K, and a protease inhibitor cocktail were purchased from Wako Pure Chemical Corp. (Osaka, Japan). Mineral oil, 3-Azido-1-propanol, and calcein were purchased from Merck (Darmstadt, Germany). N-succinimidyl-S-acetylthioglycolate (SATA), hydroxylamine hydrochloride, DBCO-maleimide, and Methyl-PEG_24_-Azide were purchased from Tokyo Chemical Industry (Tokyo, Japan). Amplex Red Phospholipase D Assay Kit and Alexa Fluor 633 succinimidyl ester were purchased from Thermo Fisher Scientific (Waltham, MA, USA). PUREfrex 2.0 was purchased from GeneFrontier, Inc. (Chiba, Japan). All aqueous solutions were prepared with ultrapure water from a Milli-Q system (Milli-Q Integral 3, Merck KGaA, Darmstadt, Germany).

### Preparation of the lipid vesicles and observation of Spo20-GFP fluorescence on the lipid vesicles

Each 100 µL of 1 mM DOPC/1 mM DOPA (3.7:0.3, 9:1, 17:3, 4:1, and 7:3 molar ratio) dissolved in chloroform was added to a glass test tube. The lipid solution was evaporated under flowing argon gas until lipid films formed at the bottom of the test tube. The lipid films were hydrated using 100 µL of buffer (10 mM HEPES, 140 mM NaCl, and 500 mM sucrose, pH 7.4) and incubated for over 12 h at 27 °C. 5 mL of the lipid vesicles were added to 43.56 µL of buffer (10 mM HEPES, 140 mM NaCl, and 500 mM glucose, pH 7.4) and 1.44 µL of 1.74 µM Spo20-GFP (final concentration: 50 nM). After incubation for 60 min at 37 °C, Spo-GFP was observed in the vesicles using a confocal laser scanning microscope (CLSM) (FV1200, Olympus, Tokyo, Japan) with an oil-immersion lens (×60) and using a diode laser (473 nm) for GFP. The fluorescence intensity of GFP on the lipid vesicle membrane was measured using Image J (NIH, Bethesda, MD, USA). The fluorescence intensity of GFP on the lipid vesicle membrane was measured by the line profile.

### Synthesis of phospholipase D (PLD) using cell-free protein synthesis system (CFPS)

We synthesized PLD using two kinds of CFPS (PUREfrex 2.0 and *E. coli* extract S30). In the case of PUREfrex 2.0, the reaction mixture (Solution I 10 µL, Solution II 1 µL, Solution III 2 µL, Template DNA [100 ng/µL] 1 µL, and Milli-Q water 7 µL) was incubated for 4 h at 37 °C. In the case of *E. coli* extract S30, the reaction mixture (LMCPY-tRNA 11.2 µL; 17.5 mg/mL tRNA 0.3 µL: 5% NaN_3_ 0.3 µL; 1.6 M Mg[OAc]_2_, 0.28 µL; 20 mM each amino acid 2.25 µL; 3.75 mg/mL Creatine Kinase 2 µL; 10 mg/mL T7 RNA polymerase 0.2 µL; S30 extract 9 µL; plasmid DNA [final concentration: 30 ng/µL) 3 µL; and Milli-Q water 1.47 µL) was incubated for 4 h at 37 °C.

### Preparation of S30 extract

An S30 extract obtained according to a method published in a previous paper^61^. Rosetta2 (DE3) pLysS was grown on the LB agar plates containing 25 μg/mL chloramphenicol overnight. Colony of Rosetta2 (DE3) pLysS was cultured in 30 mL LB medium containing 25 μg/mL chloramphenicol and grown in a 100 mL baffled flask for 16 h at 37 °C and 200 rpm. Next, 30 mL of growth medium was diluted to 570 mL of 2× YT+P+G medium (7 g/L potassium phosphate dibasic, 3 g/L potassium phosphate monobasic, and 1.8% glucose (final concentration)). The cultures were grown for approximately 4 h at 37 °C and 200 rpm to OD_600_ = approximately 3. The cultures were centrifuged for 15 min at 5000 × *g* and 4 °C. The cell pellets were washed three times with washing buffer (50 mM Tris, 14 mM Mg-glutamate, 60 mM K-glutamate, 2 mM DTT) on ice. The pellets were centrifuged between each wash step for 15 min at 5000 × *g* and 4 °C, and finally centrifuged for 15 min at 5000 × *g* and 4 °C. The pellets were frozen using liquid nitrogen and stored at −80 °C. Next day, the pellets were defrosted on ice. Pellets were suspended in 1 mL washing buffer/ g pellet. The solution was sonicated using a probe-type sonicator at a duty cycle of 80% and 30% amplitude by 10 s ON/OFF for 20 times (Sonifer 250A, Branson, Brookfield, CT). The solution was centrifuged for 10 min at 12,000 × *g* and 4 °C. The supernatant was collected into the new tube. The tube was covered with aluminum foil and shaking at 37 °C and 200 rpm for 80 min. The solution was centrifuged for 10 min at 12,000 × *g* and 4 °C. The supernatant was dialyzed using dialysis buffer (5 mM Tris, 14 mM Mg-glutamate, 60 mM K-glutamate, 1 mM DTT, pH 8.2) at 4 °C for 3 h. After dialysis, the solution was centrifuged for 10 min at 12,000 × *g* and 4 °C. The supernatant (extract) was aliquoted into a new tube on ice. The extract was frozen using liquid nitrogen and stored at −80 °C.

### Enzyme activity assay of PLD

The enzyme activity of PLD was confirmed using the Amplex Red Phospholipase D Assay Kit. One µL of the reaction mixture synthesizing PLD was diluted with 99 µL of reaction buffer (250 mM Tris-HCl, 25 mM CaCl_2_, pH 8.0). The diluted solution was added to 100 µL of the working solution containing Amplex red, horseradish peroxidase (HRP), Choline oxidase, and L-α-Phosphatidylcholine. 571 nm of absorbance of time lapse was measured using a microplate reader (Synergy H1 Multimode Reader). The final concentration of DTT in the measurement solution was approximately 2 µM.

### Synthesis and enzyme activity of PLD under the control of riboswitch

A PLD-corded DNA was inserted into a pJBL3752 plasmid vector, which was a gift from Julius Lucks (Addgene plasmid #128809; http://n2t.net/addgene:128809; RRID:Addgene_128809), for controlling translation and transcription in the presence of sodium fluoride (NaF). We used *E. coli* extract S30 for synthesizing PLD in the presence of NaF. The reaction mixture (LMCPY-tRNA 7.48 µL; 17.5 mg/mL tRNA 0.2 µL: 5% NaN_3_ 0.2 µL; 1.6 M Mg(OAc)_2_, 0.18 µL; 20 mM each amino acid 1.5 µL; 3.75 mg/mL Creatine Kinase 1.32 µL; 10 mg/mL T7 RNA polymerase 0.14 µL; S30 extract 6 µL; NaF (final concentration: 0 and 5 mM) 0.5 µL; plasmid DNA (final concentration: 20 ng/µL) 1 µL; and Milli-Q water 1.48 µL) was incubated for 4 h at 37 °C. After incubation, these reaction mixtures were stored at 4 °C.

### Conversion of PC to PA inside lipid vesicles using PLD synthesized by cell-free synthesis systems

The giant lipid vesicles were formed by a droplet transfer method as follows. The phospholipid DOPwas dissolved in chloroform and transferred into 4 mL glass tubes. The tubes were then placed in a vacuum desiccator for 1 h. A dry lipid film formed on the glass tubes. Mineral oil was added to those films, and the mixture was sonicated for 99 min at 50 °C. The final lipid concentration in mineral oil was 1.1 mM. The lipid solution was stored at 4 °C. To obtain a water-in-oil (w/o) emulsion including cell-free synthesis systems, 12.5 µL of 1× LMCPY, 0.175 mg/mL tRNA, 0.05% NaN_3,_ 15 mM Mg (OAc)_2_, 1.5 mM each amino acid, 0.25 mg/mL creatine kinase, 67 ng/µL T7 RNA polymerase, 30% S30 extract, 50 nM Spo20-GFP, 10 ng/µL plasmid were added to 100 µL of the lipid solution in an Eppendorf tube and vortexed for 2 min. These components in the Eppendorf tube were emulsified by hand-tapping the tube. A thin lipid monolayer was formed at the interface between 30 µL of the lipid solution and 30 µL of HEPES-glucose buffer (10 mM HEPES, 75 mM NaCl, and 500 mM glucose; pH 7.4). The w/o emulsion (50 µL) was added to the oil phase of the thin lipid monolayer. Finally, the solution was centrifuged to transfer the w/o emulsion through the lipid monolayer at 6900 × *g* for 10 min at 4 °C. After centrifugation, the giant lipid vesicles that accumulated at the bottom of the tube were collected (15 µL). The giant lipid vesicles were diluted using 5 µL of the outer solution (LMCPY; 0.05% NaN_3_; 15 mM Mg(OAc)_2_; 1.5 mM each amino acid; S30 buffer; and Milli-Q water). 3 mM NaF at final concentration or Milli-Q. After incubation for 1 h at 37 °C, Spo-GFP was observed in the vesicles using a confocal laser scanning microscope (CLSM) (FV1200, Olympus, Tokyo, Japan) with an oil-immersion lens (×60) and using a diode laser (473 nm) for GFP. The fluorescence intensity of GFP on the lipid vesicle membrane was measured using Image J (NIH, Bethesda, MD, USA) (Supplementary Fig. 4).

### Confirmation of asymmetric lipid vesicle formation using the hydrolysis activity of PLD synthesized by cell-free synthesis systems under the control of riboswitch

The phospholipids DOPC and DOPC/cholesterol (2:1 molar ratio) were dissolved in chloroform and transferred to 4 mL glass tubes. The tubes were then placed in a vacuum desiccator for 1 h. A dry lipid film formed on the glass tubes. Mineral oil was added to those films, and the mixture was sonicated for 99 min at 50 °C. The final lipid concentration in mineral oil was 1.1 mM. These lipid solutions were stored at 4 °C. To obtain a water-in-oil (w/o) emulsion including cell-free synthesis systems, 12.5 µL of 1× LMCPY, 0.175 mg/mL tRNA, 0.05% NaN_3_, 15 mM Mg (OAc)_2_, 1.5 mM each amino acid, 0.25 mg/mL creatine kinase, 67 ng/µL T7 RNA polymerase, 30% S30 extract, 50 nM Spo20-GFP, 10 ng/µL plasmid, and milliQ water (for observation of PA in the inner leaflet) or 1× LMCPY, 0.175 mg/mL tRNA, 0.05% NaN_3_, 15 mM Mg (OAc)_2_, amino acids at 1.5 mM each, 0.25 mg/mL creatine kinase, 67 ng/µL T7 RNA polymerase, 30% S30 extract, 10 ng/µL plasmid, and milliQ water (for observation of PA in the outer leaflet) were added to 100 µL of the lipid solution in an Eppendorf tube and vortexed for 2 min. These components in the Eppendorf tube were emulsified by hand-tapping the tube. A thin lipid monolayer was formed at the interface between 30 µL of the lipid solution and 30 µL of HEPES-glucose buffer (10 mM HEPES, 75 mM NaCl, and 500 mM glucose; pH 7.4). The w/o emulsion (50 µL) was added to the oil phase of the thin lipid monolayer. Finally, the solution was centrifuged to transfer the w/o emulsion through the lipid monolayer at 6900 × *g* for 10 min at 4 °C. After centrifugation, the giant lipid vesicles that accumulated at the bottom of the tube were collected (15 µL). The giant lipid vesicles were diluted using 30 µL of the outer solution (LMCPY; 0.05% NaN_3_; 15 mM Mg(OAc)_2_; 1.5 mM each amino acid; S30 buffer; and Milli-Q water (for observation of PA in the inner leaflet) or LMCPY; 0.05% NaN_3_; 15 mM Mg(OAc)_2_; 1.5 mM each amino acid; S30 buffer; 50 nM Spo20-GFP; and Milli-Q water (for observation of PA in the outer leaflet)). NaF at a final concentration of 3 mM was added to the outer solution of the vesicles. After incubation for 15 min, 30 min, 45 min, 60 min, 75 min, and 90 min at 37 °C, Spo20-GFP on the vesicle membranes was observed using CLSM with an oil-immersion lens (×60) and using a diode laser (473 nm) for GFP. The fluorescence intensity of GFP on the lipid vesicle membrane was measured using Image J (Supplementary Fig. 4).

### Conjugation of alkyne to purified mCherry

The pET-28a/mCherry with a 6xHis tag at the C-terminus was transfected into E. coli strain BL21(DE3). Cultures were grown at 37 °C in a Lysogeny broth (LB) medium containing 20 µg/mL kanamycin to OD_600_ = 0.8. Protein expression was induced by adding isopropylthio-β-galactoside at a final concentration of 0.5 mM to the culture medium and shaking at 37 °C for 4 h. The culture medium was then centrifuged at 3000 × *g* for 20 min at 4 °C. The collected cell pellets were stored at –80 °C. The cell pellets were resuspended in a buffer solution (TE buffer (50 mM Tris, 1 mM ethylenediaminetetraacetic acid (EDTA), pH 7.2) containing 5 mg/mL lysozyme and 500 µg/mL DNase I). After incubation for 10 min at room temperature, the TE buffer was added to the cell suspension. The cell suspension was incubated for 10 min at –80 °C, and the frozen cell suspension was thawed at room temperature. The cell suspension was centrifuged at 7000 × *g* for 20 min at 4 °C, and the supernatant was collected. The supernatant and Ni-NTA agarose resin equilibrated with buffer solution (10 mM Tris, 140 mM NaCl, pH 7.4) were mixed for 1 h at 4 °C. After washing the resin with 20 mM Tris, 300 mM NaCl, 5 mM imidazole, pH 8.0, mCherry was eluted with 20 mM Tris, 300 mM NaCl, 200 mM imidazole, pH 8.0. The purified mCherry was concentrated using an ultrafiltration (Amicon ultracentrifugal filter (MWCO 10 kDa)), and the protein concentration was measured using a Nanodrop One (Thermo Fisher Scientific) with coefficients of molar absorbance at 280 nm. The proteins were stored at −80 °C. The mCherry solution was exchanged to 100 mM phosphate buffer (pH 7.2) containing 150 mM NaCl using the ultrafiltration. N-succinimidyl S-acetylthioacetate (SATA) and mcherry were mixed at 50:1 molar ratio and reacted for 30 min at room temperature. To remove the unreacted SATA, the reaction solution was exchanged to 100 mM phosphate buffer containing 150 mM, pH 7.2 using ultrafiltration. The deacetylation solution (100 mM phosphate buffer (pH 7.2) containing 500 mM hydroxylamine hydrochloride, 150 mM NaCl, and 25 mM EDTA) was added to the mCherry solution to deprotect the sulfhydryl groups. After the reaction for 3 h at room temperature, the thiolated mCherry was purified to 100 mM phosphate buffer (pH 7.2) containing 150 mM NaCl using ultrafiltration. The mixture of DBCO-maleimide and thiolated mCherry at 10:1 molar ratio was reacted for 17 h at 4 °C. The unreacted DBCO-maleimide was removed from this solution using ultrafiltration.

### Accumulation of mCherry on the vesicle membrane under the control of NaF riboswitch

DOPC/cholesterol (1.1 mM; 2:1 in a molar ratio) dissolved in mineral oil was prepared. The solution compositions in the inner phase and outer phase of the lipid vesicles for the accumulation of mCherry were as follows: 1× LMCPY, 0.175 mg/mL tRNA, 0.05% NaN_3,_ 15 mM Mg (OAc)_2_, 1.5 mM each amino acid, 0.25 mg/mL creatine kinase, 67 ng/µL T7 RNA polymerase, 30% S30 extract, 1.25 µM DBCO-conjugated mCherry, 10 ng/µL plasmid, and milliQ water (inner solution) and LMCPY; 0.05% NaN_3_; 15 mM Mg(OAc)_2_; 1.5 mM each amino acid; S30 buffer; and Milli-Q water. After centrifugation, 15 µL of the lipid vesicles were collected at the bottom of the tube. The outer solution was added to the lipid vesicle solution. NaF at a final concentration of 3 mM was added to the outer solution of the vesicles. After incubation for 1 h at 37 °C, the mCherry on the lipid vesicle membranes was observed using CLSM with an oil-immersion lens (×60) and a diode laser (559 nm) for mCherry excitation. The fluorescence intensity of mCherry on the lipid vesicle was measured using Image J (Supplementary Fig. 4).

### MscL purification

MscL was purified from the *E. coli* strain BL21(DE3) transformed with the pET28a/MscL plasmid. The purification of MscL was performed according to the method followed by Baba et al.^53^. *E. coli* BL21(DE3) (Nippon Gene, Tokyo, Japan) transfecting pET28a/MscL with a 6xHis tag at the C-terminus were grown for 16 h at 37 °C in LB medium containing 20 µg/mL kanamycin to OD_600_ = 0.8-1. Isopropylthio-β-galactoside at a final concentration of 1 mM was added to the culture medium and shaken at 37 °C for 4 h. The culture was centrifuged for 20 min at 3000 × *g* and 4 °C. The cell pellets were resuspended in a buffer solution (50 mM Tris, 200 mM NaCl (pH 8.0)) containing 15.4 mM LDAO (pellets/buffer = 1 g:10 mL) and sonicated on ice using a Sonifer 250A (Branson, Brookfield, CT) after adding a small amount of DNase I. The supernatant was centrifuged for 40 min at 3500 × *g* and 4 °C. After centrifugation, the supernatant and TALON metal affinity resin were mixed for 1 h at 4 °C. The MscL was eluted with 20 mM HEPES, 20 mM NaCl, 240 mM imidazole, and 1.75 mM LDAO (pH 7.5). The purified MscL was concentrated using an ultrafiltration (Amicon ultracentrifugal filter (MWCO 10 kDa)).

### Function control of MscL transport under the control of NaF riboswitch

The DOPC vesicles containing the CFPS system and plasmid DNA were generated using the method described in this paper. After centrifugation, 15 µL of the lipid vesicles were collected at the bottom of the tube. 5 mL of the outer solution was added to the solution of the lipid vesicles. 1 mL of 92.3 µM MscL was added to the lipid vesicle solution. This concentration of MscL was chosen in accordance with our previous studies^53^. After incubation for 10 min at 4 °C, 0.36 µL of 200 mM NaF (final concentration: 3 mM), 2.18 µL of 110 µM LysoPC (final concentration: 10 µM), and 0.46 µL of 52.2 µM calcein (final concentration: 1 µM) were added to the lipid vesicle solution. After incubation for 30 min or 1 h at 37 °C, calcein in lipid vesicles was observed using CLSM with an oil-immersion lens (×60) and a diode laser (473 nm). The fluorescence intensity of calcein into the lipid vesicles was measured using image J.

### Statistics and reproducibility

All data were analyzed using Excel and OriginPro 2020b. Data are shown as mean ± SD. For all experiments, the sample sizes and number of replicates were mentioned in figure captions. Statistical analysis was performed using two-sided Student’s *t*-test. *p*-values < 0.01 were considered significant. Data in Fig. 4 are presented as box-and-whisker plots. The box represents the interquartile range (25th–75th percentiles), with the median indicated by a horizontal line. Whiskers extend to the 5th and 95th percentiles. The mean is shown as a dot.

## Supplementary information

Supplementary information

Supplementary Data 1

Description of Additional Supplementary Files

## Data Availability

Source data for all the graphs is provided in Supplementary Data 1. All other data is available from the corresponding author upon reasonable request.
