# Supplementary material for: Riboswitch-controlled lipid conversion enables functional membrane asymmetry in artificial cells
Source: Commun Biol. 2026 Mar 19;9:580. doi: 10.1038/s42003-026-09890-7 (PMC13121617; doi:10.1038/s42003-026-09890-7)
Supplement: Supplementary file 1 — Supplementary information [file 42003_2026_9890_MOESM1_ESM.pdf]

SUPPLEMENTARY INFORMATION

Riboswitch-Controlled Lipid Conversion Enables Functional Membrane Asymmetry in Artificial Cells

Koki Kamiya [1]*, Sumin Lee [1], Kotaro Baba [1]

Graduate School of Science and Technology, Gunma University, Gunma 376-8515, Japan

* **Corresponding author:** Koki Kamiya

1-5-1 Tenjin-cho, Kiryu,

Gunma, Japan

Tel: +81-277-30-1342

Email: kamiya@gunma-u.ac.jp

DOPC vesicles: inner		max.	min.		max. mol%	min. mol%
	90min	6322.267	2018.364		19.31779764	11.47106603
	75min	6961.933	1669.067		20.18665187	10.51799216
	60min	8636.048	1979.421		22.27325097	11.36950085

DOPC vesicles: outer		max.	min.		max. mol%	min. mol%
	90min	3399.32	1717.333		14.55279277	10.6557579
	75min	8638.111	1717.087		22.27567958	10.65506111
	60min	no signal	no signal		no signal	no signal

DOPC/chol vesicles: inner		max.	min.		max. mol%	min. mol%
	90min	9027.062	4740.133		22.72805417	16.93794251
	75min	7005.417	1970.427		20.24410922	11.34589007
	60min	7886.385	1683.059		21.36887059	10.55814994

DOPC/chol vesicles: outer		max.	min.		max. mol%	min. mol%
	90min	1627.73	no signal		10.39827236	no signal
	75min	1941.73	no signal		11.27016141	no signal
	60min	no signal	no signal		no signal	no signal

Supplementary Table 1. The extent of PA flop (PA translocation from the inner leaflet to the outer leaflet) on lipid vesicles was estimated based on the fluorescence intensities in Figure 4 and the calibration curve shown in Supplementary Figure 6, which allows conversion of fluorescence intensities above 10 mol% PA.

The amount of PA in the inner or outer leaflet was calculated from the fluorescence intensity within the 5% to 95% confidence interval. No signal represents the fluorescence intensities below 10 mol% PA.

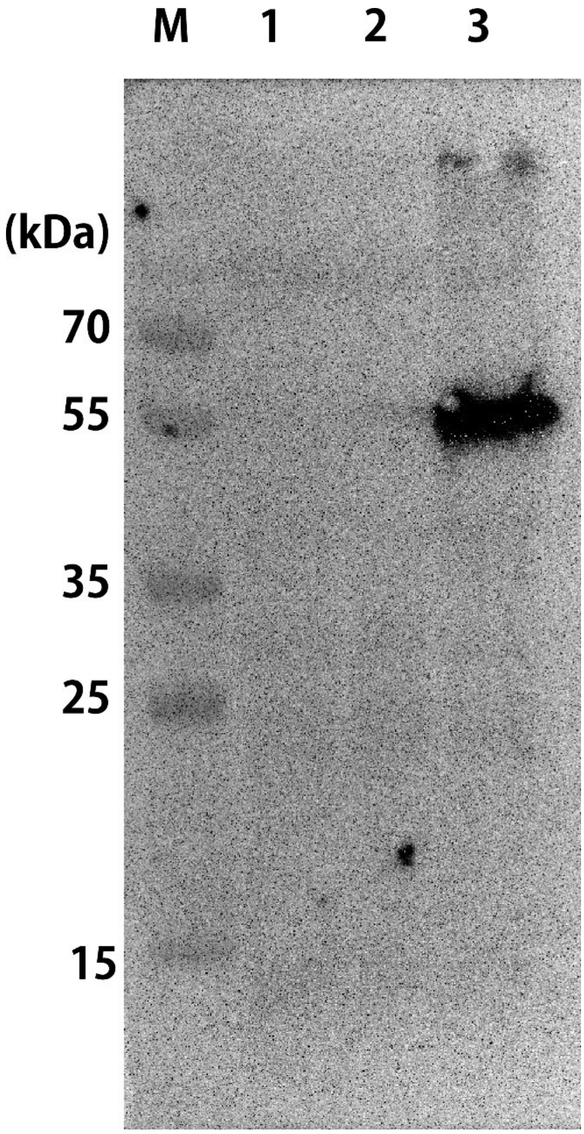

Supplementary Figure 1. Full-length western blot analysis for Figure 2 (a).

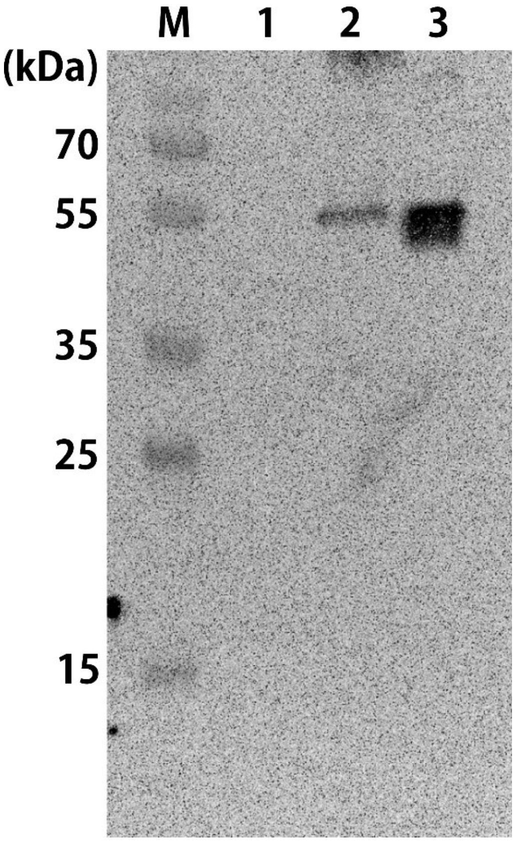

Supplementary Figure 2. Full-length western blot analysis of PLD synthesized by PUREfrex 2.0. M: protein marker; Lane 1: only PUREfrex 2.0 solution (−DNA), Lane 2: wild-type PLD; Lane 3: mutant PLD

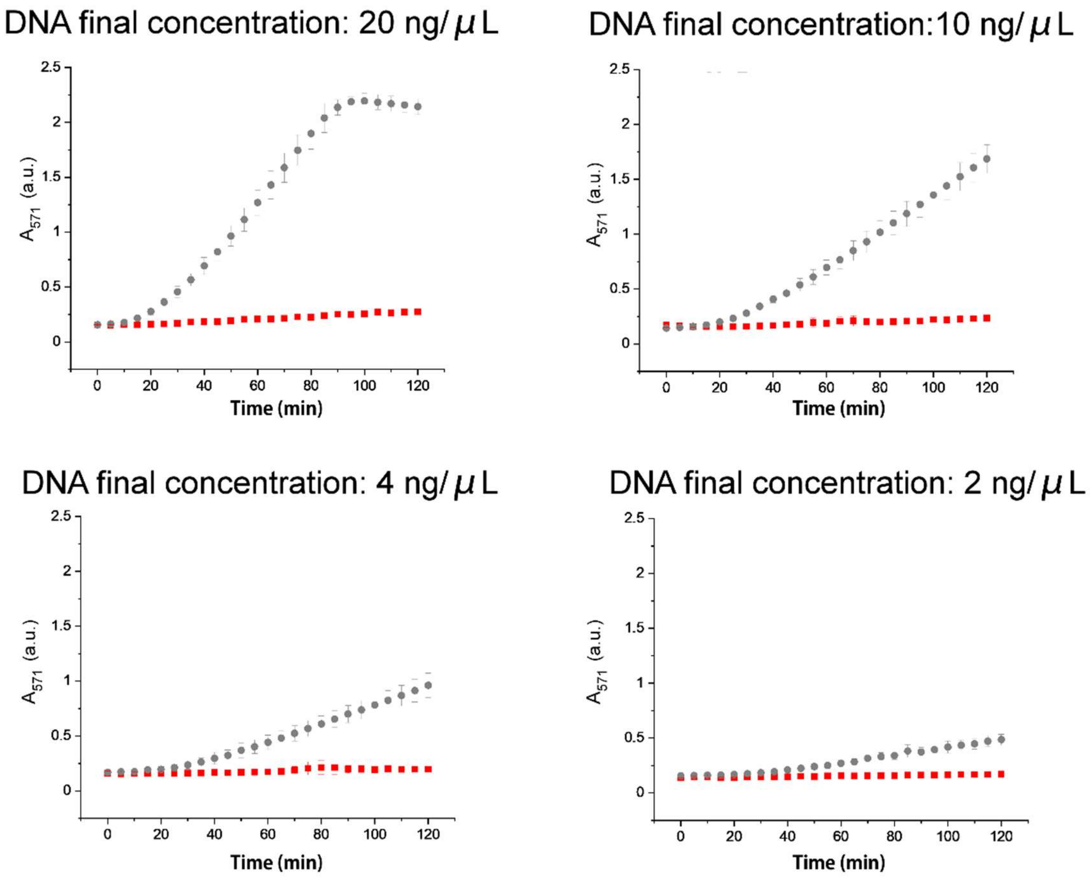

Supplementary Figure 3. Mutant PLD synthesized by fluoride-responsive riboswitch of the activity assay of choline hydrolysis chaining 20 ng/μL, 10 ng/μL, 4 ng/μL, and 2 ng/μL of plasmid DNA final concentration (gray: +DNA and +NaF; orenge: +DNA and −NaF) (N= each 3 experiment). Three mM NaF was added to the microtube.

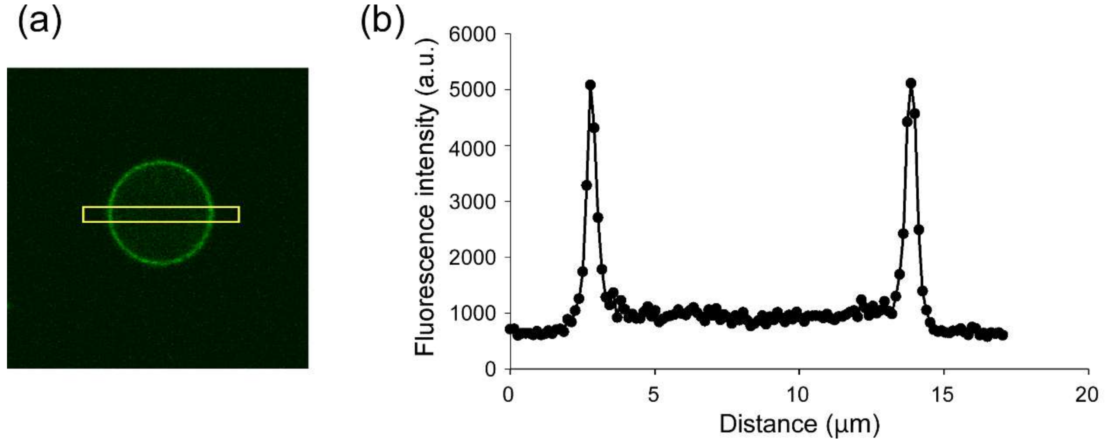

Supplementary Figure 4. Method for measuring the fluorescence intensity on the membrane by ImageJ. (a) Location of measuring (b) line plot of fluorescence intensity of this location.

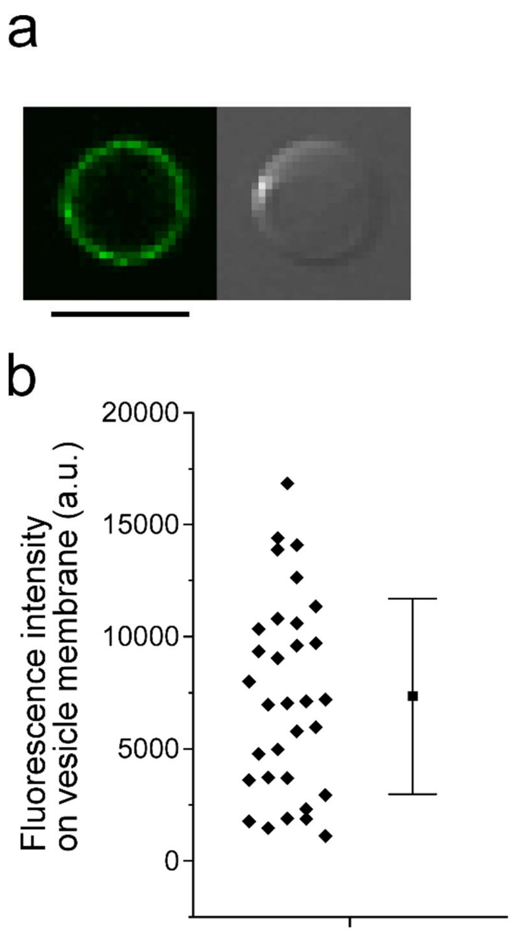

Supplementary Figure 5. Image (a) and fluorescence intensity (b) on 20 mol% DOPA vesicle membranes. The vesicles containing 20 mol% DOPA in both leaflets were formed by the gentle hydration method. Spo20-GFP was added to the outer solution of the vesicles (N= each 3 experiment). After incubation for about 10 min, the vesicles were observed by CLMS. Scale bar, 5 μm.

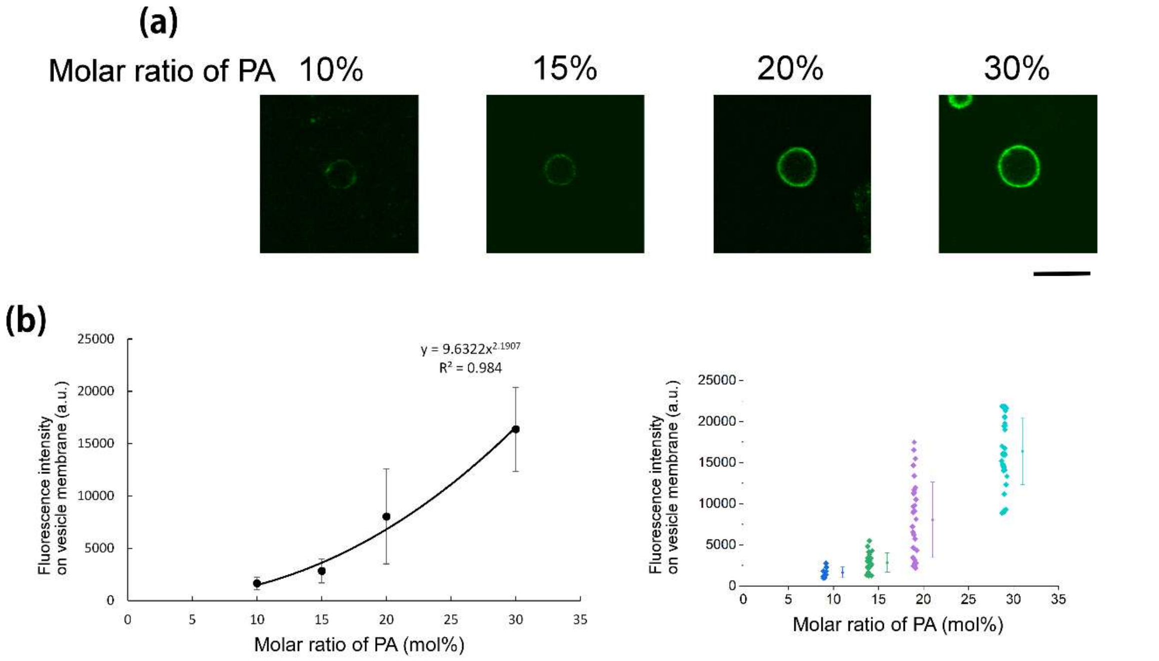

Supplementary Figure 6. (a) Typical confocal images of GUVs containing 10 mol% to 30 mol% of DOPA. Spo20-GFP was added to the outer solution of the vesicles. After incubation for about 10 min, the vesicles were observed by CLMS. (b, left) Calibration curve of fluorescence intensity on vesicle membranes and molar ratio of PA in the outer leaflet. Scale bar: 10 μm. Lipid vesicles formed by a gentle hydration method contained 10 mol% (n = 8 vesicles), 15 mol% (n = 24 vesicles), 20 mol% (n = 31 vesicles), and 30 mol% (n = 35 vesicles) of DOPA in both leaflets. Error bars represent the standard deviation. (b, right) Dot plot of fluorescence intensity on vesicle membranes and molar ratio of PA in the outer leaflet; 10 mol% (n = 8 vesicles), 15 mol% (n = 24 vesicles), 20 mol% (n = 31 vesicles), and 30 mol% (n = 35 vesicles) of DOPA in both leaflets. Error bars represent the standard deviation.

We observed the EGFP fluorescence on the membrane of the 7.5 mol% PA vesicles. The EGFP fluorescence from some vesicles containing 7.5 mol% PA was observed. On the other hand, EGFP fluorescence was observed from all vesicles containing 10 mol% PA. In this experiment condition, we found that over 10 mol% PA of the vesicles can be detected. Therefore, this calibration curve is only used to convert over 10 mol% PA of the vesicles. Bars represent the standard deviation.

The fluorescence intensity increased with PA concentration, showing a gradual rise at 10–15 mol% PA followed by an approximately proportional increase at 20–30 mol%. This behavior may be attributed to a surface charge-dependent membrane binding process, where a threshold PS density is required for efficient protein association. The PA concentration dependence was fitted using an exponential function as an empirical model to describe the observed nonlinear trend within the experimental range.

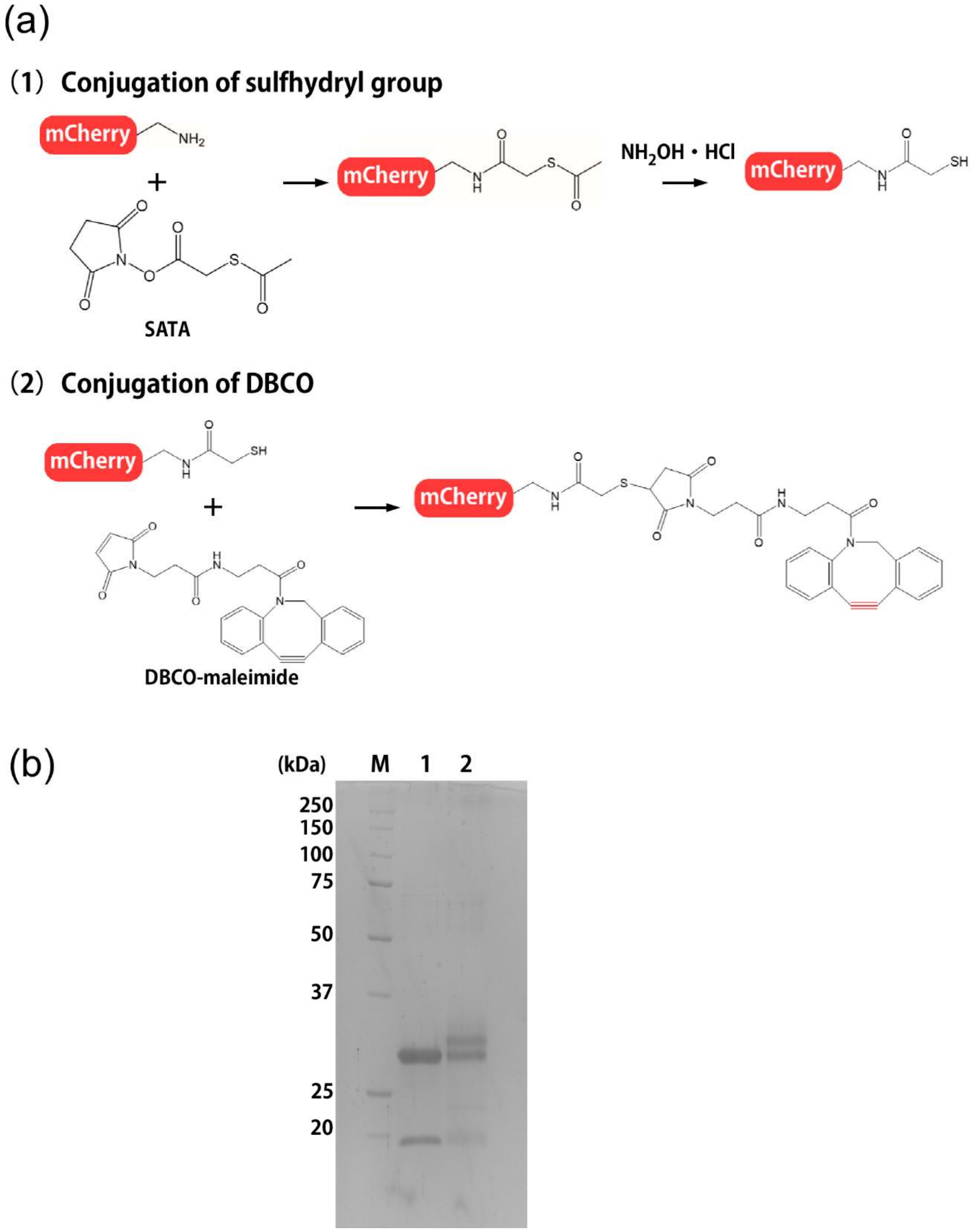

Supplementary Figure 7. (a) scheme of DBCO-conjugated mCherry. (b) SDS-PAGE analysis for confirming the conjugation of DBCO to mCherry. M: Marker, Lane 1: mCherry, Lane 2: DBCO-conjugated mCherry.

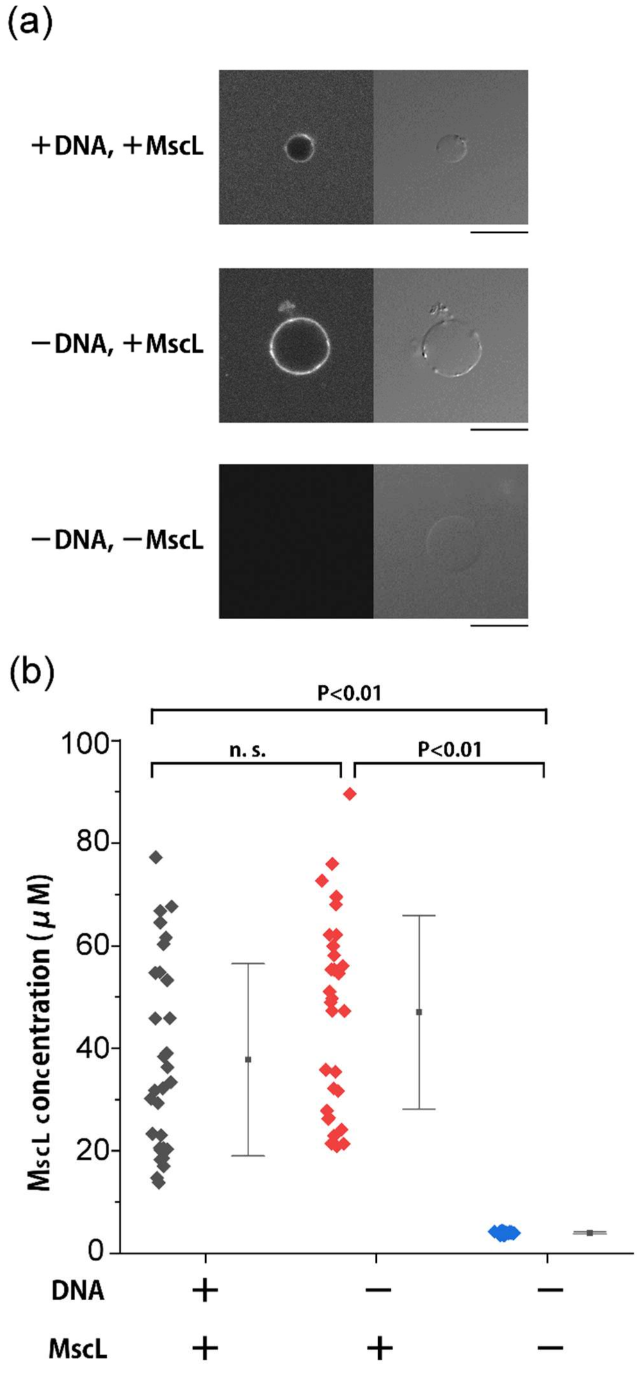

Supplementary Figure 8. (a) Confocal laser scanning microscopy fluorescence images of the lipid vesicles with/without MscL. Scale bar, 10 um. (b) Concentration of MscL on the lipid vesicles with DNA and MscL (n=30 vesicles, N= 3 experiments), with MscL (No DNA) (n=30 vesicles, N= 3 experiments), and without DNA and MscL (n=30 vesicles, N= 3 experiments).

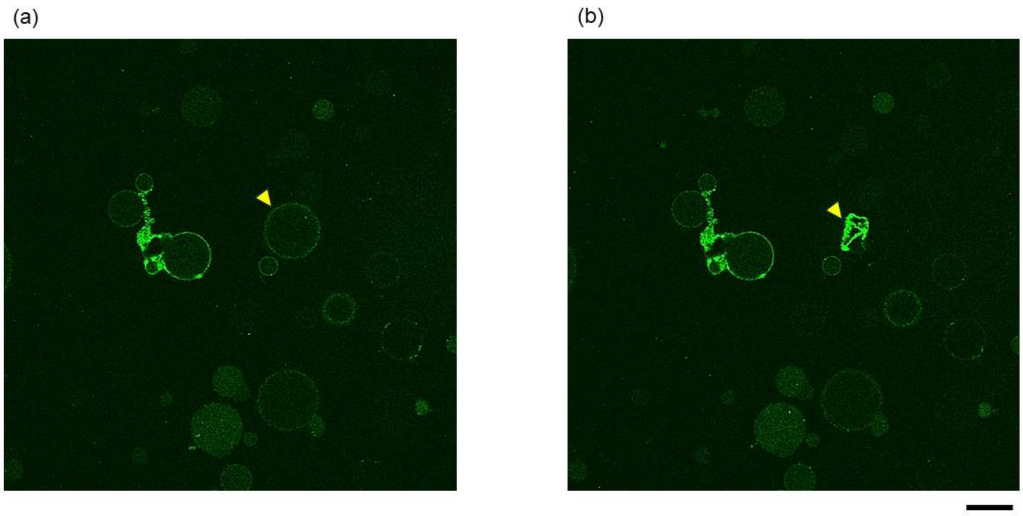

Supplementary Figure 9. Time lapse images of the DOPC vesicles containing Spo20-GFP synthesized the mutant PLD (a) and after 5 min of the lipid vesicles (b). A yellow triangle shows collapse of the vesicle. Scale bar: 10 μm.
