# Supplementary material for: Riboswitch-controlled lipid conversion enables functional membrane asymmetry in artificial cells
Source: Commun Biol. 2026 Mar 19;9:580. doi: 10.1038/s42003-026-09890-7 (PMC13121617; doi:10.1038/s42003-026-09890-7)
Supplement: Supplementary file 3 — Description of Additional Supplementary Files [file 42003_2026_9890_MOESM3_ESM.docx]

**Description of Additional Supplementary File**

File name: Supplementary data 1
Description: Source data for all the graphs is provided in Supplementary Data 1
